# Supplementary material for: Host and Seasonal Effects on the Abundance of Bean Leaf Beetles (Ootheca spp.) (Coleoptera: Chrysomelidae) in Northern Uganda
Source: Insects. 2022 Sep 18;13(9):848. doi: 10.3390/insects13090848 (PMC9503690; doi:10.3390/insects13090848)
Supplement: Supplementary file 1 [file insects-13-00848-s001.zip › insects-1835633-supplementary.pdf]

Table S1: Delineated growth stages for common bean, cowpea and soybean

Growth stage	Description
1. Seedling stage	• VC – two cotyledons and primary leaves visible at nodes 1 and 2, respectively<br>• V1 – first tri-foliate leaf unfolded at node 3<br>• V2 – second tri-foliate leaf unfolded at node 4<br>• V3 – third tri-foliate leaf unfolded at nod 5
2. Vegetative with branching	• V4 – forth tri-foliate leaf unfolded at node 6 + branching<br>• Vn – more than four tri-foliate leaves, without open flower
3. Flowering and pod formation	• R1 – at least one open flower on the plant<br>• R2 – Fifty percent open flowers<br>• R3 – One pod at maximum length<br>• R4 – fifty percent of pods at maximum length
4. Pod maturation	• R5 – one pod with fully developed seeds<br>• R6 – fifty percent of pods with fully developed seeds<br>• R7 – one pod at mature colour (physiological maturity)<br>• RH – eighty percent of pods at mature colour (harvest maturity)

Table S2: Degrees of freedom, F statistics (v.r.), and p-value (F pr.) from repeated measures analysis of variance for number of *Ootheca* spp., leaf damage, and percent plants with beetles

		Transformed number of BLBs per plot		Leaf damage		Transformed percent plants with BLBs	
Source of variation	d.f.(m.v.)	v.r.	F pr.	v.r.	F pr.	v.r.	F pr.
Replication stratum	3	0.52		0.87		2.02	
Replication x Subject stratum							
Host plant	2	75.14	<.001	254.78	<.001	85.10	<.001
Season	2	816.83	<.001	194.26	<.001	1224.69	<.001
District	1	546.62	<.001	194.21	<.001	725.31	<.001
Host plant x Season	4	53.54	<.001	10.60	<.001	28.22	<.001
Host plant x District	2	37.66	<.001	51.27	<.001	22.26	<.001
Season x District	2	470.36	<.001	54.71	<.001	448.66	<.001
Host plant x Season x District	4	26.02	<.001	6.37	<.001	6.95	<.001
Residual	51	1.17		2.22		0.62	
Replication x Subject x time stratum d.f. correction factor 0.4888							
Time	8	20.53	<.001	111.01	<.001	13.98	<.001
Time x Host plant	16	3.24	0.002	9.05	<.001	1.35	0.200
Time x Season	16	19.55	<.001	32.35	<.001	13.44	<.001
Time x District	8	16.35	<.001	43.18	<.001	10.99	<.001
Time x Host plant x Season	32	2.79	<.001	3.14	<.001	0.94	0.536
Time x Host plant x District	16	3.64	<.001	7.77	<.001	2.21	0.016
Time x Season x District	16	14.44	<.001	31.20	<.001	7.78	<.001
Time x Host plant x Season x District	32	3.15	<.001	6.41	<.001	1.62	0.046
Residual	430(2)						
Total	645(2)						

Table S3: Degrees of freedom, F statistic (v.r.), and p-value (F pr.) repeated measures analysis of variance for abundance of below-ground life stages of *Ootheca* spp.

Source of variation	d.f.	Transformed number of larvae per plot		Transformed number of pupae per plot		Transformed number of below-ground adults per plot	
		**v.r.**	**F pr.**	**v.r.**	**F pr.**	**v.r.**	**F pr.**
Rep stratum	3	0.60		1.09		3.55	
Rep x Subject stratum							
Host plant	2	23.23	<.001	13.24	<.001	17.88	<.001
Season	2	105.58	<.001	24.36	<.001	35.18	<.001
District	1	18.86	<.001	0.02	0.887	13.72	<.001
Host plant x Season	4	14.42	<.001	7.81	<.001	6.35	<.001
Host plant x District	2	1.23	0.302	0.07	0.934	1.07	0.351
Season x District	2	31.95	<.001	1.77	0.181	10.24	<.001
Host plant x Season x District	4	3.93	0.007	0.77	0.553	1.83	0.137
Residual	51	0.87		2.64		1.46	
Rep x Subject x Time stratum<br>d.f. correction factor 0.3920							
Time	8	17.20	<.001	2.18	0.072	5.52	<.001
Time x Host plant	16	6.51	<.001	0.72	0.676	2.24	0.021
Time x Season	16	19.71	<.001	2.39	0.018	14.62	<.001
Time x District	8	4.40	0.005	0.89	0.472	2.80	0.022
Time x Host plant x Season	32	6.53	<.001	1.11	0.351	3.48	<.001
Time x Host plant x District	16	2.75	0.013	0.64	0.740	0.93	0.498
Time x Season x District	16	3.25	0.004	0.48	0.865	3.65	<.001
Time x Treatment x Season x District	32	2.38	0.006	0.46	0.963	0.87	0.617
Residual	432						
Total	647						

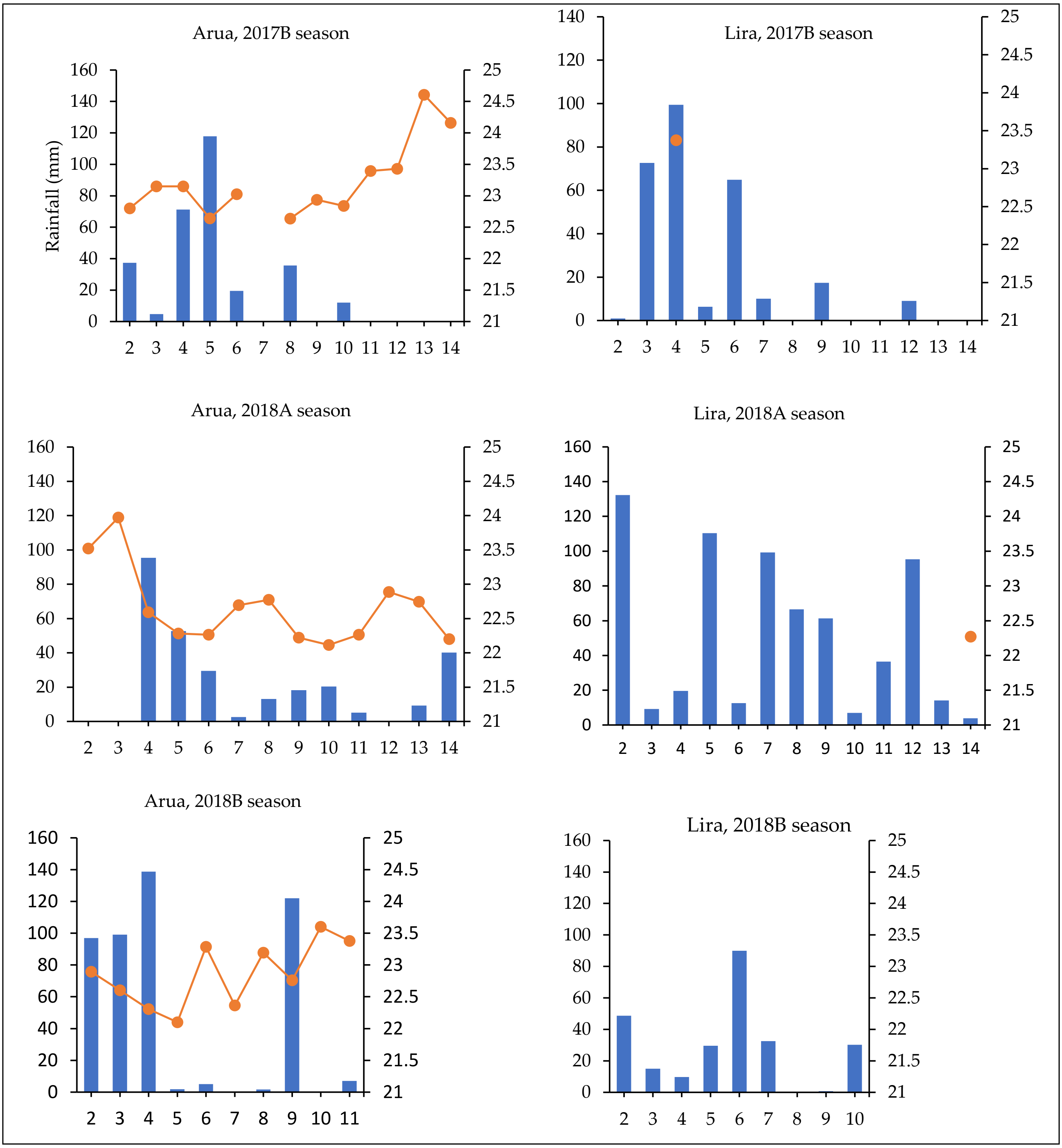

Figure S1: Rainfall and temperature distribution against weeks after planting in Arua and Lira during 2017B, 2018A and 2018B rain season. Temperature data is missing for many points in Lira district due to breakdown of devices. Metrological data source is Uganda Metrological Authority (UMA)
